# Starch-Based Hydrogels as a Drug Delivery System in Biomedical Applications

**DOI:** 10.3390/gels9120951

**Published:** 2023-12-04

**Authors:** Chung-Sung Lee, Hee Sook Hwang

**Affiliations:** 1 Department of Pharmaceutical Engineering, Soonchunhyang University, Asan 31538, Republic of Korea; 2 Department of Pharmaceutical Engineering, Dankook University, Cheonan 31116, Republic of Korea

**Keywords:** starch, hydrogel, drug delivery, tissue engineering, biomedical application

## Abstract

Starch-based hydrogels have gained significant attention in biomedical applications as a type of drug delivery system due to their biocompatibility, biodegradability, and ability to absorb and release drugs. Starch-based hydrogels can serve as effective carriers for pharmaceutical compounds such as drugs and proteins to develop drug-loaded hydrogel systems, providing controlled release over an extended period. The porous structure of a hydrogel allows for the diffusion of drugs, ensuring sustained and localized delivery to the target site. Moreover, starch-based hydrogels have been used as a powerful option in various biomedical fields, including cancer and infectious disease treatment. In addition, starch-based hydrogels have shown promise in tissue engineering applications since hydrogels can be used as scaffolds or matrices to support cell growth and tissue regeneration. Depending on techniques such as chemical crosslinking or physical gelation, it can create a three-dimensional network structure that tunes its mechanical properties and mimics the extracellular matrix. Starch-based hydrogels can also provide a supportive environment for cell attachment, proliferation, and differentiation to promote specific cellular responses and tissue regeneration processes with the loading of growth factors, cytokines, or other bioactive molecules. In this review, starch-based hydrogels as a versatile platform for various biomedical applications are discussed.

## 1. Introduction

Starch is a major source of carbohydrate and the most abundant storage polysaccharide in plants. It has been widely explored in the pharmaceutical and biomedical industries due to its biocompatibility and degradability. In addition, it is considered a non-toxic and non-immunogenic material. Starch can be prepared from different sources, such as corn, potatoes, and rice, while maintaining its biocompatible properties. Starch is a mixture of two polymers: amylose and amylopectin, which are monosaccharides or glucose molecules linked via α-D-(1-4) and/or α-D-(1-6) linkages [1]. Amylose, a linear polysaccharide of glucose units linked via α-1-4 glycosidic bonds, has a molecular mass of 107–109 g/mol. It accounts for 20–30% of the starch composition [2]. Amylose forms inclusion complexes with linear, polar, and nonpolar compounds, including fatty alcohols, long-chain fatty acids, and monoglycerides. Amylopectin has chain lengths of 20–25 glucose units. It is a branched macromolecular component. It forms glucose via α-1-6 glycosidic bonds. It constitutes 70–80% of the starch composition [3].

Starch-based hydrogels have emerged as a promising class of biomaterials with significant potential for various biomedical applications. Hydrogels are three-dimensional (3D) networks of hydrophilic polymers that can absorb and retain large amounts of water or biological fluids while maintaining their structural integrity [4,5]. Starch has unique properties such as biocompatibility, biodegradability, and the ability to absorb and release drugs, making it an excellent candidate for the development of hydrogel systems in the field of biomedicine [6]. For instance, hydrogels can be applied to artificial skin, contact lenses, blood contact materials, the interface between bones, and implants. Due to its controlled release properties, it can be applied for the delivery of anticoagulants, contraceptives, enzymes, and hormones [6,7,8]. 

Starch-based hydrogels have garnered considerable attention as drug carriers due to their ability to encapsulate a wide range of drugs and proteins, allowing for controlled release over an extended period [6,9,10]. The porous structure of these hydrogels enables the diffusion of drugs, ensuring their sustained and localized delivery to the target site. This property is particularly advantageous in the treatment of various diseases, including cancer. This is because site-specific drug delivery can enhance therapeutic efficacy while minimizing systemic side effects. In addition, starch-based hydrogels have shown promise in the field of tissue engineering by serving as scaffolds or matrices to support cell growth and tissue regeneration [7,11]. Depending on the fabrication technique, starch-based hydrogels can create a 3D network structure that mimics the natural extracellular matrix, providing a supportive environment for cell attachment, proliferation, and differentiation. By incorporating bioactive molecules, starch-based hydrogels can further enhance specific cellular responses and biomedical processes.

**Figure 1:**
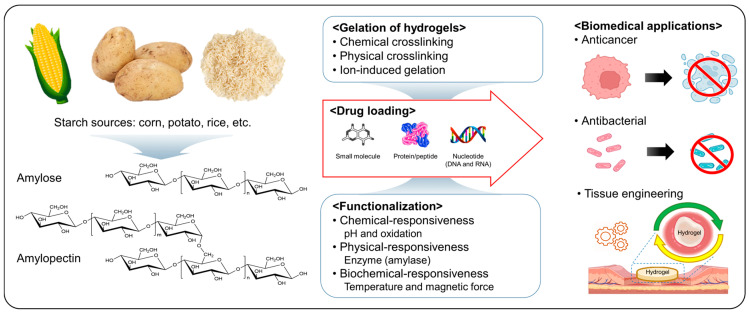
Starch-based hydrogels as drug delivery systems in biomedical applications. This schematic diagram was created with BioRender.com.

Overall, this review will summarize the crosslinking techniques employed in the development of starch-based hydrogels. Their use as drug delivery systems is also explored, highlighting their ability to encapsulate and release pharmaceutical compounds, their controlled release mechanisms, and their applications in targeted therapies. Furthermore, we will delve into the current state of biomedical applications of starch-based hydrogels (Figure 1). Especially, this review discusses the biomedical applications of starch-based hydrogels, based on a search performed through Google Scholar and PubMed using the keywords starch, hydrogel, drug delivery, tissue engineering, and biomedical application. We hope that this review will contribute to the understanding and advancement of starch-based hydrogels as versatile biomaterials.

## 2. Starch-Based Hydrogels as Drug Delivery Systems

### 2.1. Types of Pharmaceutical Compounds

Starch-based hydrogels have been extensively explored as delivery systems for a wide range of pharmaceutical compounds. The selection of pharmaceutical compounds for loading into starch-based hydrogels depends on factors such as drug properties, therapeutic targets, and desired release profiles. It is essential to consider the physicochemical compatibility between the hydrogel matrix and loaded compounds to ensure stability and sustained release. Various techniques, including physical mixing, covalent conjugation, and encapsulation within micro- or nano-particles, can be employed to incorporate these compounds into the hydrogel matrix [12].

Moreover, starch-based hydrogels offer versatility in the types of pharmaceutical compounds that can be loaded for drug delivery applications. Pharmaceutical compounds that can be encapsulated within starch-based hydrogels are diverse, ranging from small molecules to proteins, bioactive natural compounds, and combination therapies, opening up opportunities for various therapeutic applications.

**Figure 2:**
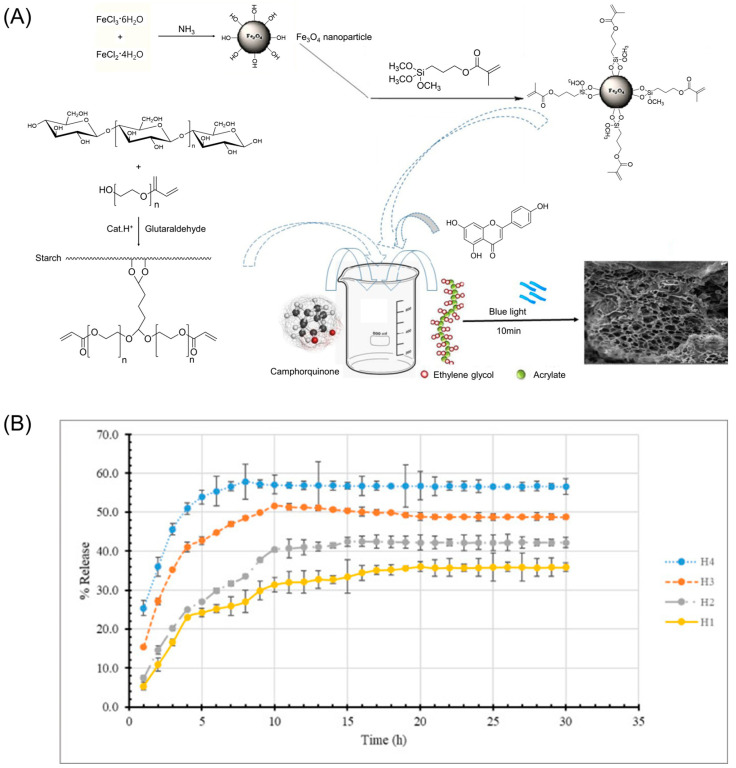
Quercetin-loaded starch-based nanocomposite hydrogels. (**A**) Schematic illustration of the preparation of starch-based nanocomposite hydrogels with Fe_3_O_4_ nanoparticles and quercetin. (**B**) In vitro quercetin release profile from starch-based nanocomposite hydrogels with different swelling degrees: 103.52% for H1, 110.40% for H2, 125.46% for H3, and 140% for H4. Reproduced with permission from Moghadam et al. [14] (Copyright 2022, Elsevier B.V.).

Starch-based hydrogels can effectively encapsulate small-molecule drugs, including antibiotics, analgesics, and anti-cancer drugs. The hydrogel matrix provides a protective environment for these small molecules, preventing degradation and enhancing their stability during storage and release. An anticancer drug (DOX) can be delivered by a starch-based stimuli-responsive magnetite nanohydrogel [13]. Moghadam et al. have developed a biodegradable starch-based hydrogel for delivering quercetin, an important drug in cancer treatment (Figure 2) [14]. Quercetin is a drug that is poorly water-soluble, has low permeability, and has poor oral bioavailability. Moghadam et al. have prepared a starch-based hydrogel modified by polyethylene glycol/acrylate and Fe_3_O_4_ nanoparticles to improve the release rate of quercetin. The hydrogel was found to enhance in vitro drug release to ~57% after 8 h.

Kamoun [15] has prepared N-succinyl chitosan (SCS)–dialdehyde starch hybrid (DAS) hydrogels without using any chemical crosslinkers and loaded curcumin, which is known to be an anti-tumor agent having anti-inflammatory and anti-oxidant properties. These hydrogels were non-toxic, biodegradable, and biocompatible. They showed various physicochemical properties by changing the SCS:DAS ratio in hydrogels, which resulted in differences in gelation time, equilibrium swelling, morphological structure, mechanical stability, and the in vitro release profile of curcumin.

Xu et al. [16] have designed starch/microcrystalline cellulose hybrid gels by ionic liquid dissolution and regeneration to produce a continuous surface, a porous structure, and a crystalline structure of cellulose for a gastric-floating drug delivery system. These hybrid gels were loaded with ranitidine hydrochloride as a model drug. They prepared ranitidine hydrochloride capsules, which showed excellent floating performance and sustained release behavior that improved the bioavailability of ranitidine hydrochloride.

Most conventional antibiotics, such as penicillin G, have been loaded in a starch-citrate hydrogel as a carrier for antibiotics for infection treatments [17]. In addition, guaifenesin delivery via starch-based nanocomposite hydrogel has been investigated [18]. This nanocomposite hydrogel can release the drug in external magnetic field- and pH-dependent manners.

Starch-based hydrogels have been explored for the encapsulation of bioactive natural compounds such as plant extracts, essential oils, and phytochemicals. These natural compounds often possess therapeutic properties. They have various applications, including wound healing, anti-inflammatory treatments, and antimicrobial therapies. The hydrogel matrix enables the controlled release of these compounds, prolonging their therapeutic effects. Ghaffar et al. [19] have prepared a poly(starch/acrylic acid) (1:10 wt%) hydrogel for delivering rutin, a citrus flavonoid glycoside found in many plants, for anti-inflammatory and antiallergic treatment. An in vitro release study showed that the hydrogel had a pH-dependent release behavior. An in vivo study demonstrated that rutin-loaded hydrogel inhibited colonic inflammation with reduced toxicity. Thus, they have concluded that such a pH-sensitive hydrogel is an effective anti-inflammatory approach to maximize the therapeutic effect of rutin for inflammatory diseases.

Furthermore, proteins such as growth factors, cytokines, and antibodies can be loaded into a hydrogel matrix to provide sustained release and localized delivery [20,21,22]. Komur et al. [21] have designed injectable hydrogels composed of sodium tetraborate, polyvinyl alcohol, and starch-based hydrogels to deliver bone morphogenic protein-2 (BMP-2). Since protein and/or peptide growth factors have a short half-life, they aimed to prolong the half-life of BMP-2 and increase its bioactivity to cure bone injuries. Controlled release of BMP-2 from the hydrogel enhanced the proliferation of healthy cells and alkaline phosphatase (ALP) activity in bone cells compared to the control group without hydrogel. Thus, the hydrogel showed promise for targeted drug therapies. Faikrua et al. [20] have studied a thermosensitive chitosan/starch/β-glycerol phosphate hydrogel as a delivery carrier for chondrocytes and transforming growth factor-βI (TGF-β). They incorporated growth factor and chondrocyte into the gel and demonstrated that the system could stimulate chondrocyte function and secretion of ECMs, aggrecan, and type II collagen for cartilage tissue engineering.

### 2.2. Controlled Release Mechanisms and Kinetics

Controlled release is a crucial aspect of drug delivery systems. Starch-based hydrogels offer versatile mechanisms to achieve controlled release of pharmaceutical compounds. Release kinetics from these hydrogels can be adjusted by modulating several factors, including hydrogel composition, crosslinking density, drug-loading strategies, and environmental stimuli. Understanding controlled release mechanisms and kinetics is essential for designing efficient drug delivery systems using starch-based hydrogels.

Diverse mechanisms are involved in the controlled drug release of starch-based hydrogels. These mechanisms include diffusion-controlled release, swelling-controlled release, degradation-controlled release, and stimulus-responsive release. By understanding and optimizing these mechanisms, the release kinetics of starch-based hydrogels can be modulated, enabling precise control over drug delivery profiles and enhancing therapeutic outcomes.

One of the primary mechanisms of drug release from starch-based hydrogels is diffusion. Drug molecules can diffuse through the hydrogel matrix from regions of high concentration to regions of lower concentration until an equilibrium is reached. The release rate depends on various factors, such as the diffusion coefficient of the drug within the hydrogel, the concentration gradient, the hydrogel matrix density, and the hydrogel porosity. Particularly, the porous structure of hydrogels plays a crucial role in diffusion-controlled release. The hydrogel network forms interconnected pores that enable the diffusion of drug molecules. By controlling the porosity and pore size of the hydrogel, the release rate can be adjusted. Increasing hydrogel porosity generally leads to faster drug release, whereas reducing the pore size can slow down the release rate.

Reis et al. [23] have designed a chemically modified starch (starch-M) with glycidyl methacrylate and demonstrated that the water transport mechanism of the hydrogel is dependent on Fickian diffusion, which is controlled by water diffusion. The purpose of designing the hydrogel was to transport and preserve drugs responsive to an acidic environment. They suggested that starch-M hydrogel could be used for transporting acid-responsive drugs such as corticoids for treating colon diseases. Xu et al. [16] have used starch and microcrystalline cellulose hybrid gels to generate gastric-floating drug delivery systems with a diffusion-controlled release. These hybrid gels were loaded with ranitidine hydrochloride as a model drug. Rapid release of the drug from the hybrid tablet was observed in the initial stage due to the weak diffusion resistance of tablets and the fact that ranitidine hydrochloride molecules were mainly released through the interior network structure of hybrid tablets by diffusion. They found that such starch and microcrystalline cellulose hybrid gels showed diffusion-controlled drug release as gastric-floating drug delivery systems.

Most starch-based hydrogels are highly hydrophilic. They can absorb and retain a significant amount of water or biological fluids. When a hydrogel absorbs water, it swells, leading to changes in its physical and chemical properties. Swelling can influence drug release by two mechanisms: diffusion through the swollen hydrogel matrix and desorption from the hydrogel surface. In the case of diffusion through the matrix, the swollen hydrogel creates a hydrated environment that can facilitate drug diffusion. The release rate is influenced by the swelling capacity of the hydrogel, whose swelling capacity depends on various factors such as crosslinking density, hydrogel composition, and environmental conditions such as pH and temperature.

Desorption from the surface of a hydrogel occurs when drug molecules are not fully entrapped within the hydrogel matrix but are adsorbed or bound to the surface. As the hydrogel swells, drug molecules near the hydrogel surface are released due to desorption. The release rate can be controlled by modifying the hydrogel’s surface properties, such as surface charge and hydrophilicity.

Elvira et al. [6] have developed starch-based biodegradable hydrogels via free radical polymerization by mixing both a solid and a liquid component to generate thermoplastic and crosslinked hydrogels with a desirable kinetic behavior for a controlled release system. Swelling kinetics were determined as a function of pH in different buffer solutions. It was found that these hydrogels exhibited pH-sensitive, degradable, and swelling characteristics that could be used in a range of biomedical applications. In addition, a new cross-linked starch-xanthan gum hydrogel has been developed for use as a film-forming material in controlled-release drug delivery formulations [24]. The reaction of starch-xanthan gum polymers with sodium trimetaphosphate determines the swelling properties and gel mesh size of the hydrogel film. The swelling ratio of the film was higher at higher contents of sodium trimetaphosphate and xanthan gum. The gel mesh size increased with an increasing swelling ratio, which determined the release kinetics of drugs. The hydrogel also showed selective permeability depending on drug charges, which could be useful for designing controlled releases of ionizable drugs.

Moreover, a new Fe_3_O_4_/starch-*g*-poly(ethylene phthalate) hydrogel nanocomposite has been prepared by grafting the copolymerization of poly(ethylene phthalate) onto starch [25]. The structure of the hydrogel nanocomposite and the relationship of Fe_3_O_4_ content with swelling behavior were determined [25]. When water absorption in nanocomposite with different amounts of Fe_3_O_4_ was determined, it was found that concentrations of 0.01 M FeCl_2_ and 0.005 M FeCl_3_ resulted in the maximum water absorption and swelling. Thus, the nanocomposite without Fe_3_O_4_ nanoparticles was the most suitable formulation for drug delivery since it had the best drug loading capacity and releasing behavior [25].

Starch-based hydrogels can be engineered to respond to external stimuli such as temperature, pH, light, and magnetic fields to achieve controlled drug release. These stimulus-responsive hydrogels undergo structural changes in response to specific stimuli, resulting in the release of encapsulated drugs.

Temperature-responsive hydrogels exhibit a lower critical solution temperature (LCST) or an upper critical solution temperature behavior. Below or above this critical temperature, the hydrogel undergoes a transition, leading to changes in its swelling behavior and drug release kinetics. By selecting appropriate temperature-sensitive polymers or modifying the composition of the hydrogel, the release of drugs can be triggered or controlled by temperature changes. Cardoso et al. [26] have developed hydrogels of gellan gum and starch retrograded blends for drug delivery applications by controlling polymer and cross-linker concentrations. Structural modifications of the retrograded starch blend hydrogels were made using isothermal cycles at 4 °C or alternating thermal cycles. At cooling conditions (4 °C/8 days), hydrogels formed a more organized and stable structural network that resulted in a harder and more cohesive material. Hydrogels formed a looser network with higher mobility and became more adhesive under cycled temperatures (4 °C and 30 °C/16 days). In addition, Cardoso et al. [26] have demonstrated that these hydrogels can be adjusted for suitable adhesiveness, high strength, and elasticity as promising materials for mucoadhesive drug delivery systems. Dong et al. [27] have also prepared a starch-based thermosensitive hydrogel that shows reversible swelling and deswelling behavior as the temperature changes. They demonstrated that the addition of hydroxybutyl starch and PEG generated more porous structures and increased the swelling ratio. In addition, these hydrogels exhibited repeated swelling at 25 °C and deswelling at 45 °C.

It is known that pH-responsive hydrogels can take advantage of pH differences between different body compartments or disease conditions. By incorporating pH-sensitive moieties such as acid-labile or base-labile linkages within hydrogels, the release of drugs can be tailored to specific pH environments. It is known that pH-responsive starch-based hydrogels can enable targeted drug delivery to acidic tumor microenvironments or specific regions within the body. Gholamali et al. [28] have prepared oxidized starch/CuO nanocomposite hydrogels prepared in situ during CuO nanoparticle formation in an oxidized starch hydrogel matrix for application in a drug delivery system. When the swelling behavior was investigated at pH 2.1 and 7.4, these nanocomposite hydrogels showed a pH-sensitive swelling behavior. The swelling ratio at pH 2.1 was lower than that at pH 7.4 because the conversion of carboxyl groups on oxidized starch chains to negatively charged carboxylate ions resulted in electrostatic repulsion. These oxidized starch/CuO nanocomposite hydrogels showed a higher swelling ratio than pure oxidized starch hydrogels. The degree of swelling of the hydrogels is determined by charged CuO nanoparticles that can cause expansion of the hydrogel network with an increased number of pores and free spaces within the hydrogel matrix. As a consequence, these hydrogels exhibited sustained and controlled drug release depending on CuO nanoparticle content in a pH-dependent manner. In addition, a pH-sensitive magnetic biomaterial has been developed by grafting the polymerization of itaconic acid onto starch and alginate in the presence of graphene sheets and Fe_3_O_4_ nanoparticles for drug delivery [29]. The increase in starch and alginic acid content significantly enhanced drug release. Moreover, the hydrogel possessed a magnetic property. The use of an external magnetic field demonstrated a positive correlation with the rate of drug release. When a magnetic field was applied, there was a greater release of drugs. Due to such pH dependence and sustained release behavior, a platform was proposed for magnetically targeted drug delivery [29].

Starch-based stimuli-responsive magnetite nanohydrogels (MNHGs) have been developed for targeted delivery of doxorubicin [13]. These hydrogels were designed using magnetite nanoparticles since they could target the desired area through external magnets. They showed no magnetic interaction after removing external magnetic fields. These hydrogels also displayed dual (pH and temperature)-responsive drug release behavior [13]. DOX-loaded MNHG showed a higher drug release at 42 °C and pH 5.3 than at pH 7.4 and 37 °C. It was explained that such release behavior might result from the degradation of PNIPAAm [poly(N-isopropylacrylamide)] in hydrogels at higher temperatures that resulted in the release of DOX [14]. Furthermore, Silva et al. [30] have developed starch graft copolymers by physical blending of Am-MA (amylose starch-methacrylic acid) and Am-HEMA (amylose starch-2-hydroxyethyl methacrylate) that offer different properties of permeability, pH sensitivity, and biodegradability to develop matrices for colon-specific drug delivery. They found that using a matrix mixture of Am-MA and Am-HEMA copolymers provided a controlled release of the drug, which was primarily controlled by Am-HEMA. In addition, they observed that there were release differences between matrix formulations in acidic and basic media since swelling of the matrix had different drug release profiles.

**Figure 3:**
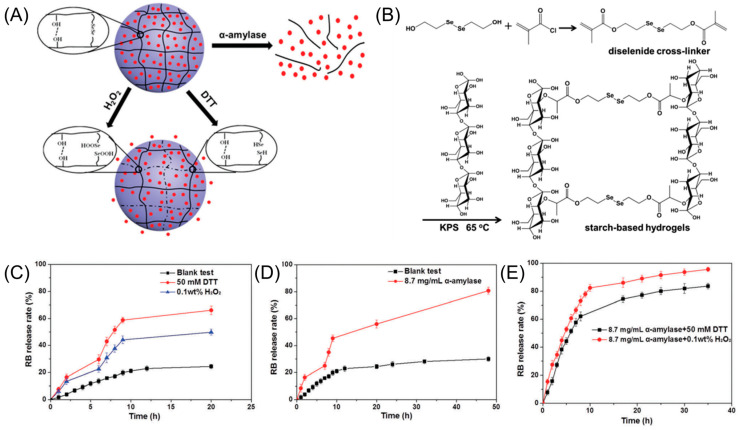
Dual stimuli-responsive selenium-functionalized starch-based hydrogels. Red dot=Rhodamine B. (**A**) Schematic illustration of stimuli-responsive starch-based hydrogels by enzyme, oxidation, and reduction responsiveness. (**B**) Synthetic routes of stimuli-responsive starch-based hydrogels. KPS = potassium persulfate. (**C**–**E**) Rhodamine B release profiles of starch-based hydrogels under various conditions. DTT = dithiothreitol. Reproduced with permission from Sun et al. [31] (Copyright 2018, Royal Society of Chemistry).

Enzymatic degradation is particularly relevant in biological environments where specific enzymes can degrade a starch-based hydrogel matrix. By incorporating enzymatically cleavable bonds or crosslinkers within the hydrogel, the degradation rate can be tuned, leading to controlled drug release. Enzyme-responsive starch-based hydrogels have been explored for targeted delivery to specific tissues or cells that possess relevant enzymes. Sun et al. [31] have designed and synthesized biodegradable crosslinked starch-based hydrogels via free radical polymerization, as shown in Figure 3. They designed stimuli-responsive drug release material using enzyme hydrolysis properties and a selenium-containing cross-linker with a redox-responsive cleavage property. Such starch-based hydrogels showed controlled release in redox conditions and fast release in α-amylase solution due to enzyme hydrolysis of starch backbone degradation. Thus, these hydrogels composed of enzyme- and redox-responsive properties demonstrated great potential applications in drug delivery.

## 3. Starch-Based Hydrogels in Biomedical Applications

Starch-based hydrogels have shown immense potential in targeted therapies across various biomedical applications ranging from cancer-targeted therapy to ophthalmic drug delivery, infectious disease treatment, regenerative medicine, and tissue engineering. These hydrogels offer versatile platforms for efficient biomedical applications.

Starch-based hydrogels have emerged as promising platforms for cancer therapy due to their versatility and ability to incorporate various anti-cancer drugs. These hydrogels can enhance therapeutic efficacy while reducing the adverse effects of free drugs. Additionally, stimulus-responsive starch-based hydrogels can be utilized to achieve site-specific drug release in response to the tumor microenvironment, such as an acidic pH or overexpressed enzymes.

Saboktakin et al. [32] have fabricated a starch-based hydrogel using carboxymethyl starch (CMS) and dextran sulfate (DS) to encapsulate a porphyrin-based photosensitizer (PS) agent for the photodynamic treatment of cancer. A carboxymethyl starch (CMS) hydrogel using dextran sulfate (DS) as a polyanionic polymer showed a high degree of protection from premature drug release and allowed controlled release of PS molecules at desired sites. Thus, they insisted that such hydrogel could be further applied for cancer treatment, with expected accumulation at tumor sites followed by site-specific degradation and drug release.

A biodegradable starch nanocrystal/gum Arabic hydrogel has been developed for controlled drug delivery and cancer therapy [33]. Starch nanocrystals were prepared by the acid hydrolysis of starch. They were used to produce a starch nanocrystal/gum Arabic blend with the addition of potassium persulfate (K_2_S_2_O_8_) as an initiator. In vitro anticancer activity assays of this hydrogel demonstrated that the cell death rate was dependent on the rate of drug release from the hydrogel in a human colorectal cancer cell line [33].

Wang et al. [34] have developed a biocompatible iodine-starch-alginate (ALG) hydrogel for tumor photothermal therapy. They introduced photothermal therapy (PTT) for high efficiency with minimal invasiveness for tumor therapy. Such hydrogel displayed biocompatibility derived from its biosafe and degradable components and good photothermal heating ability generated via an iodine-starch chromophore. In vivo PTT studies demonstrated a significant tumor-suppressing effect of the iodine-starch-ALG hydrogel on tumor-bearing mice after photothermal treatment. No tumor recurrence was observed in the hydrogel group at 14 days after treatment compared to the control group, demonstrating the excellent PTT capability of the hydrogel.

Cancer photothermal therapy using injectable thermosensitive iodine-loaded starch-g-poly(N-isopropylacrylamide) hydrogel has been performed by Wang et al. [35]. These thermosensitive nanogels were composed of iodine-loaded starch grafted with poly(N-isopropylacrylamide). They were used as PTT materials and bactericides. These nanogels displayed a sol-gel phase transition at a physiological temperature and displayed strong photothermal and anti-infection effects. In vitro studies demonstrated that these hydrogels not only killed tumor cells under laser irradiation but also suppressed bacteria effectively. An in vivo study demonstrated that tumors were completely suppressed by these hydrogels on day 14, with no relapse after treatment. Thus, the authors insisted that these hydrogels could be applied to treat tumors and infectious diseases.

Starch-based hydrogels can be loaded with antimicrobial agents such as antibiotics or antimicrobial peptides. They can be designed to deliver these antimicrobial agents specifically to infection sites. Controlled release capabilities of starch-based hydrogels enable sustained drug release, prolonging therapeutic effects and reducing the development of drug resistance. Starch-based hydrogels can be particularly valuable in treating localized infections such as wound infections and periodontal diseases. Chin et al. [17] have prepared antimicrobial starch-citrate hydrogels by physical crosslinking of starch citrate, PVA, and PEG via the freeze-thaw technique. These starch-citrate hydrogels demonstrated outstanding antimicrobial activities against Gram-negative bacteria (such as *Escherichia coli* and *Staphylococcus pyogenes*) and Gram-positive bacteria (such as *Salmonella thypimurium* and *Streptococcus aureus*). Such antimicrobial activities of starch-citrate hydrogels could be due to the suppression of the proliferation of bacteria by disrupting their membranes. They also prepared penicillin G loaded starch-citrate hydrogel, which showed sustained release behavior over 7 days with synergistic antimicrobial activities against *E. coli* and *S. thypimurium*. Thus, these starch-citrate hydrogels are considered to be effective antimicrobial agents and drug delivery carriers.

In ophthalmic drug delivery, starch-based hydrogels can be loaded with drugs for treating ocular diseases. These hydrogels can release drugs in a controlled manner, maintaining therapeutic concentrations in ocular tissues and minimizing the need for frequent administration. Aslzad et al. [36] have developed chitosan (CS)/dialdehyde starch (DAS) hybrid in situ-forming hydrogels as novel ophthalmic delivery systems for ocular delivery of betamethasone by overcoming the ocular’s biological and physiological barriers. They demonstrated that these in situ-forming composite hydrogels provided excellent biocompatibility and hemocompatibility. Different ratios of CS to DAS affected gelation time, surface microstructure, swelling ratio, rheological behavior, and in vitro degradation. They optimized the covalent intermolecular cross-linking of polymer chains of CS and starch and proposed the efficient delivery of betamethasone. By changing the ratio of CS to DAS, gelation of hydrogels occurred in less than 1 min, indicating a desirable formulation for ocular drug delivery. A high CS to DAS ratio resulted in a fast gelation time, a high mechanical strength, a compact hydrogel structure, a low swelling ratio, and a fast drug release rate. Thus, they proposed that a higher DAS content might provide a higher potential as a successful ocular drug delivery system.

Tissue regeneration is a complex process that involves the interaction of multiple cellular and molecular factors [37]. One of the key strategies in tissue engineering is to incorporate growth factors, cytokines, and bioactive molecules into scaffolds to enhance tissue regeneration. In tissue engineering, starch-based hydrogels can serve as scaffolds or matrices to support cell growth, tissue regeneration, and controlled release of bioactive molecules. A controlled release of these bioactive molecules from a hydrogel supports the sequential activation of cellular processes, leading to the formation of functional tissues. Moreover, properties such as mechanical strength and degradation rate of starch-based hydrogels can be tailored to match the requirements of different tissues and organs.

Mao et al. [38] have investigated an oxidized starch/gelatin-based shape memory hydrogel (OSG) acting as a self-contracting wound dressing to accelerate noninvasive wound closure. The thermo-reversible gelatin acts as a shape memory switch that can enhance the structural stability and mechanical properties of OSG hydrogel. The OSG displayed an effective thermo-responsive shape memory property at 38 °C with excellent mechanical capacity as well as tissue compatibility for wound tissue remodeling. Moreover, histological analysis showed thicker epidermis and dermis layers upon OSG hydrogel treatment, suggesting that OSG facilitated tissue reconstruction in the wound area. In addition, Pal et al. [39] have designed a starch-based hydrogel by crosslinking polyvinyl alcohol with starch suspension using glutaraldehyde as a crosslinking agent. The hydrogel showed sufficient strength with reduced toxicity. In addition, the hydrogel membrane showed a tensile strength comparable to skin. Thus, it could be used as artificial skin for a wound area. They suggested that the membrane could be treated with various nutrients and healing factors at the site of action to speed up the healing process of the wound.

Cell attachment, proliferation, and differentiation are fundamental processes in tissue engineering that determine the success of tissue regeneration. Starch-based hydrogels have shown great potential for supporting these cellular activities due to their unique properties, which can create a favorable environment for cell behavior. Their biocompatibility, surface properties, porous structure, and ability to incorporate bioactive molecules make them an ideal scaffold for promoting cellular activities. By providing a suitable microenvironment, starch-based hydrogels can guide cell behavior and facilitate tissue regeneration. In this section, we will discuss the role of starch-based hydrogels in promoting cell attachment, proliferation, and differentiation.

Cell attachment to a scaffold is the initial step in tissue regeneration. It provides the foundation for subsequent cellular processes. Starch-based hydrogels offer a suitable substrate for cell attachment due to their biocompatibility and surface properties. The hydrophilic nature of starch-based hydrogels can promote cell adhesion, allowing cells to spread and establish physical interactions with the hydrogel. Dong et al. [40] have designed zwitterionic starch-based “clickable” hydrogels via a “copper- and light-free” Michael-type “thiol-ene” addition reaction for 3D cell encapsulation in hydrogel. The hydrogel mimicked and provided an ECM microenvironment. Cell-laden hydrogels demonstrated cell proliferation by increasing cell population density without causing significant cell death after 2 days. Most cells started to stretch with the budding of cells from encapsulated cells. In addition, the hydrogel effectively prevented nonspecific protein and cell adhesion, providing a blank platform for 3D cell encapsulation.

Modifications such as the immobilization of cell-adhesive peptides or proteins can provide specific ligands to promote cell-scaffold interactions. These modifications can enhance cell attachment efficiency and improve the initial cell-substrate interaction. Dong et al. [41] have developed zwitterionic starch-based hydrogels via the “thiol-ene” Michael addition reaction with CGRGDS peptide immobilized into these hydrogels to improve the specific adhesive capacity of encapsulated brown adipose-derived stem cells (BADSCs). They demonstrated that cells in such hydrogels with CGRGDS proliferated more than cells in hydrogels without CGRGDS. This observation might be due to the following facts: (1) BADSCs were adhesion cells; and (2) integrin-mediated specific cell-matrix interactions could result in cell proliferation. Thus, the introduction of CGRGCS made the starch-based hydrogel suitable for cell proliferation and “stemness” maintenance. Furthermore, Cui et al. [42] have developed an injectable starch-based tissue adhesive hydrogel composed of starch, succinic anhydride, and dopamine for hematopoiesis. Catechol groups in dopamine served as an interfacial adhesion molecule and a key component since the adhesion ability of the hydrogel was essential for bone tissue and controlled bleeding. The hydrogel showed excellent cell adhesion and proliferation with good cytocompatibility. They also confirmed that catechol groups of hydrogels enhanced cell adhesion, such as red blood cells, which could help aggregate red blood cells for better hemostatic ability.

It is important to note that cellular responses to starch-based hydrogels can be influenced by various factors, including hydrogel composition, mechanical properties, degradation rate, and the presence of bioactive molecules. Thus, careful consideration of these factors is necessary to optimize hydrogel properties for specific tissue engineering applications.

## 4. Crosslinking Techniques of Starch-Based Hydrogels

Creating a well-defined 3D network structure is crucial for the successful biomedical application of hydrogels. Various techniques can be employed to create a 3D network structure in hydrogels. Chemical crosslinking, physical crosslinking, and fabrication techniques such as freeze-drying, foaming, and electrospinning allow for the control of the hydrogel’s architecture, porosity, and mechanical properties [43,44]. The choice of technique depends on the desired properties of the hydrogel scaffold and the specific biomedical application. In this section, we will introduce several techniques that can be employed to fabricate starch-based hydrogels with desired 3D network structures.

Chemical crosslinking is a widely used technique for creating a 3D network in starch-based hydrogels [45]. It involves the formation of covalent bonds between polymer chains, resulting in a stable hydrogel structure. Chemical crosslinking agents can be added to a hydrogel precursor solution, which can then react with functional groups on starch molecules to form crosslinks. Glutaraldehyde, genipin, and carbodiimides are commonly used chemical crosslinking agents for hydrogels [46,47,48]. These agents can react with the hydroxyl groups of starch molecules, creating covalent bonds that link polymer chains together. The concentration of a crosslinking agent and the crosslinking time can be adjusted to control crosslinking density, consequently adjusting the mechanical and swelling properties of the hydrogel.

Physical crosslinking techniques rely on physical interactions rather than chemical reactions to create 3D networks in starch-based hydrogels [3,49,50]. These techniques offer advantages such as simplicity, reversibility, and the ability to encapsulate sensitive bioactive molecules. Thermo-responsive starch-based hydrogels can undergo sol-gel transitions based on temperature changes. These hydrogels are typically prepared by incorporating thermo-responsive polymers such as poly(N-isopropylacrylamide) into the starch matrix [13]. Below the LCST of a polymer, the hydrogel forms a 3D network due to hydrophobic interactions. Above the LCST, the hydrogel undergoes a sol-gel transition and becomes a solution.

By incorporating pH-responsive polymers such as poly(acrylic acid) into the hydrogel matrix, pH-sensitive starch-based hydrogels can be created [9,18]. The hydrogel swells and forms a 3D network at a specific pH range due to the ionization of acidic functional groups. Changes in pH can trigger contraction or expansion of the hydrogel, allowing for control over the 3D network structure.

Ion-induced gelation is a technique where ions are used to trigger the formation of 3D networks in starch-based hydrogels [51,52]. This method is based on ionic interactions between starch molecules and multivalent ions. By incorporating ions such as calcium and zinc ions into the hydrogel solution, ions can form bridges between starch chains, leading to the formation of a stable network structure.

Apart from crosslinking techniques, various fabrication methods can be employed to create 3D network structures of starch-based hydrogels. These techniques provide control over the architecture and pore structure of the hydrogel scaffold.

Freeze-drying, also known as lyophilization, is a commonly used technique for fabricating porous starch-based hydrogels [53,54,55]. In this process, a hydrogel precursor solution is frozen and then subjected to a vacuum, causing water to sublime. This results in the formation of a porous structure within the hydrogel, which can support cell infiltration, nutrient diffusion, and tissue integration. Xu et al. [16] have developed starch and microcrystalline cellulose hybrid gels by ionic liquid dissolution and regeneration to form a continuous surface, a porous interior, and a type-II crystalline structure of cellulose. They incorporated a drug into these gels. Low-density starch/cellulose tablets were then produced by freeze-drying under vacuum.

Foaming techniques can be used to introduce controlled porosity into starch-based hydrogels [56,57]. In this method, a gas-forming agent such as hydrogen peroxide or sodium bicarbonate is added to a hydrogel precursor solution. Upon foaming, gas bubbles create voids within the hydrogel matrix, leading to a porous structure. The size and distribution of pores can be controlled by adjusting the concentration of the gas-forming agent. Sadeghi [56] has used sodium bicarbonate as a foaming agent and prepared starch-g-poly(acrylic acid-co-2-hydroxy ethyl methacrylate) via graft copolymerization by mixing acrylic acid (AA) and 2-hydroxy ethyl methacrylate (HEMA) onto starch backbones by free radical polymerization. The porous hydrogel exhibited pH-responsiveness with swelling changes under various pHs. It showed faster release of a drug at pH 7.4 than at pH 1.2. Such swelling-deswelling behavior in acidic and basic pH environments makes these hydrogels suitable for controlled drug delivery. In addition, Kuang et al. [57] have prepared starch-based superporous hydrogels by modifying starch to acryloyloxystarch sulfate (ASS) groups and reactive vinyl groups to produce a water-soluble, biodegradable building block that has 3D networks. These starch-based superporous hydrogels were fabricated by a radical crosslinking reaction and a gas-blowing foaming process to generate a porous structure. They used PF127 and acetic acid (AA) as a foam stabilizer and a foaming aid, respectively. The time for harmonizing gelation and foaming reactions was controlled between 25 s and 60 s. The best pore structure was obtained with 30% AA and 6% ASS of DA (acryloyl group) = 5.33. It also showed fast swelling and superabsorbent properties.

Electrospinning is a technique that can be used to fabricate nanofibrous scaffolds from starch-based hydrogel precursors [58,59]. In this method, a hydrogel solution is electrostatically spun into nanofibers using a high-voltage electric field. The resulting nanofibers can form a 3D network structure with a high surface-to-volume ratio, mimicking the architecture of an extracellular matrix. Electrospinning enables the creation of scaffolds with fine-scale features that can be combined with other fabrication techniques to create hybrid structures. Hadisi et al. [58] have prepared bioactive nanobiocomposite scaffolds using silk fibroin nanofiber—starch hydrogel for bone tissue regeneration. Nanofibers were prepared by wet electrospinning in a methanol bath. Electrospun nanofibers were then incorporated into the starch matrix. Calcium phosphate was then deposited throughout the fabricated scaffolds to achieve bioactivity. They demonstrated that the morphology, structure, swelling, and calcium phosphate-forming ability could be changed by adjusting the amount of silk fibroin nanofibers in the composite. The addition of silk fibroin nanofibers to the starch matrix decreased the pore size, porosity, and swelling ratio. They also showed that increasing the amount of silk fibroin nanofibers decreased calcium phosphate formation.

Overall, Table 1 summarizes hydrogel composition, active agents, crosslinking techniques, and key findings employed in the development of starch-based hydrogels for biomedical applications.

## 5. Future Directions

This review provided a comprehensive overview of the applications of starch-based hydrogels in biomedical research. Starch-based hydrogels have gained significant attention due to their biocompatibility, biodegradability, and ability to absorb and release drugs. They have emerged as versatile platforms for drug delivery systems and tissue engineering, offering numerous advantages in these areas. Their biocompatibility ensures minimal adverse reactions and promotes the integration of hydrogels with surrounding tissues. Moreover, their biodegradability allows for gradual breakdown of the hydrogel matrix, eliminating the need for surgical removal. The ability of starch-based hydrogels to absorb and release drugs in a controlled manner is particularly valuable in drug delivery applications. This controlled release ensures sustained therapeutic effects, reduces side effects, and improves patient compliance. The versatility of these hydrogels allows for the incorporation of various pharmaceutical compounds, including small molecules and peptides. In tissue engineering, starch-based hydrogels can serve as a supportive matrix for cell growth and tissue regeneration. Their porous structure allows for nutrient and oxygen diffusion, facilitating cellular processes. Hydrogels can be tailored to mimic the mechanical properties of specific tissues, providing an optimal environment for cell attachment, proliferation, and differentiation.

Starch-based hydrogels continue to be an active area of research, with several emerging trends and potential directions for further development. Exploring the synergistic effects of combining starch-based hydrogels with other therapeutic modalities, such as phototherapy, immunotherapy, and gene therapy, can enhance treatment outcomes. This interdisciplinary approach holds promise for developing more effective and personalized treatment strategies. In addition, advancing the incorporation and controlled release of growth factors, cytokines, and other bioactive molecules within starch-based hydrogels can further enhance tissue regeneration. Targeted delivery of these molecules to specific tissues or cellular compartments can be achieved by designing stimulus-responsive hydrogels or targeted drug delivery systems. Further advancements in biofabrication techniques, such as 3D printing and bioprinting, can enable the precise fabrication of tissue-specific constructs using starch-based hydrogels. These techniques can facilitate the creation of complex structures, recapitulating the native tissue architecture. Lastly, investigating the biocompatibility and immunomodulatory properties of starch-based hydrogels is crucial for their successful translation into clinical applications. Understanding host responses to these hydrogels and developing strategies to modulate immune responses will be important for long-term implantation and tissue integration.

## 6. Conclusions

This review will summarize the crosslinking techniques employed in the development of starch-based hydrogels. Their use as drug delivery systems is also explored, highlighting their ability to encapsulate and release pharmaceutical agents, their controlled release mechanisms, and their applications in targeted therapies. Furthermore, this review highlights the significant roles of starch-based hydrogels in biomedical applications. Due to their biocompatibility, biodegradability, controlled release capabilities, and tissue engineering properties, starch-based hydrogels are versatile platforms for drug delivery systems and tissue regeneration. Moreover, various driving forces for the gelation of starch-based hydrogels, including chemical crosslinking, physical crosslinking, and several fabrication techniques, allow for the control of the hydrogel’s structure and mechanical properties. Despite certain limitations and challenges, ongoing research and advancements in the field of starch-based hydrogels are paving the way for improved performance and expanded applications. With their potential for personalized medicine, targeted therapies, and regenerative medicine, starch-based hydrogels hold promise for advancing healthcare and improving patient outcomes. In addition, we hope that this review will contribute to the understanding and advancement of starch-based hydrogels as promising biomaterials.

## Figures and Tables

**Table 1:** Composition, driving force for gelation, and key findings of starch-based hydrogels.

Hydrogel Composition (Active Agent)	Driving Force for Gelation	Key Findings	Reference
Fe_3_O_4_-*g*-[poly(*N*-isopropylacrylamide-*co*-maleic anhydride)], corn starch (doxorubicin hydrochloride)	Chemical crosslinking between the anhydride group of maleic anhydride and the hydroxyl group of starch	- Stable for more than five months at ambient conditions in phosphate-buffered saline - pH- and temperature-responsive drug release behavior of nanohydrogel	[13]
Starch-*g*-poly(ethylene glycol acrylate), silane-modified Fe_3_O_4_ (quercetin)	Chemical crosslinking between acrylate groups of starch-*g*-poly(ethylene glycol acrylate) and silane-modified Fe_3_O_4_	- Improved bioavailability (solubility and release rate) - High porosity and high-efficient drug adsorption in a modified starch hydrogel compared to an unmodified one.	[14]
N-succinyl chitosan, dialdehyde starch (curcumin)	Chemical crosslinking via Schiff’s base reaction	- Physicochemical properties (gelation time, equilibrium swelling, morphological structure, mechanical stability, and in vitro release profile) by changing chitosan:starch ratio in hydrogels	[15]
Starch (Gelose 50), Microcrystalline cellulose (PH101) (ranitidine hydrochloride)	Physical mixing, coagulation, and freeze-drying	- Gastric-floating performance and sustained release behavior	[16]
Starch, ctric acid, poly(vinyl alcohol), poly(ethylene glycol) (penicilline G)	Physical mixing, freezing, and thawing	- Excellent antimicrobial activity of starch-citrate hydrogels against gram-positive and gram-negative bacterial strains - Sustained drug release profile and synergistic antimacrobial activities with peniciline G loading	[17]
Itaconic acid-*g*-potato starch, Fe_3_O_4_ nanoparticles, (guaifenesin)	Chemical crosslinking between the carboxyl groups and Fe_3_O_4_ nanoparticles	- External magnetic field- and pH-dependent drug release - Excellent in vivo wound healing properties	[18]
Starch, acrylic acid (rutin)	Chemical crosslinking via gamma irradiation	- pH-dependent release behavior of rutin - Improved effects on anti-inflammatory evidence in a rat colitis model	[19]
Starch, poly(vinyl alcohol), sodium tetraborate (bone morphogenic protein-2; BMP-2)	Physical mixing	- Controlled release of BMP-2 - Enhanced proliferation of healthy cells and alkaline phosphatase activity in bone cells	[21]
Corn starch, chitosan, β-glycerol phosphate (transforming growth factor-β1; TGF-β1)	Physical crosslinking via the thermosensitivity of β-glycerol phosphate as a crosslinking agent	- Sustained release of TGF-β1 for 14 days - Support of chondrocyte function and chondrogenesis in the hydrogel	[20]
Corn starch, xanthan gum, sodium trimethaphosphate (FITC-dextran, vitamin B12, verapamil HCl, pyrogallol Red, Methylene Blue, caffeine, ibuprofen sodium salt, sodium salicylate)	Chemical crosslinking by sodium trimethaphosphate	- Selective gel mesh size and drug permeability depending on composition and crosslinking - Selective permeability depending on drug charges for controlled release	[24]
Starch, Fe_3_O_4_ nanoparticle, poly(ethylene phthalate) (tungstophosphoric acid)	Chemical crosslinking	- Most suitable drug loading capacity and releasing behavior in the hydrogel without Fe_3_O_4_ nanoparticles for drug delivery	[25]
High amylose starch (Hylon VII—68% amylose); gellan gum (ketoprofen)	Chemical crosslinking by aluminum chloride and glutaraldehyde	- Stronger and more stable structure in the hydrogel with higher polymer and crosslinker concentrations in the presence of drugs - Suitable adhesiveness and high strength and elasticity of the hydrogels for mucoadhesive delivery systems	[26]
Hydroxybutyl waxy corn starch, Poly(ethylene glycol) 4000 (PEG), N-isopropyl acrylamide (NIPAM)	Physical crosslinking by temperature change	- Increased microporous structure and swelling ratio with PEG and hydroxybutyl starch addition - Potential use in protein (bovine serum albumin) separation	[27]
Oxidized starch, CuO nanoparticle (39–50 nm) (ibuprofen)	Chemical crosslinking via oxidation	- Increased swelling capacity by nanocomposite - Prolonged drug release in an oxidized starch nanocomposite hydrogel	[28]
Starch, alginic acid, Fe_3_O_4_ nanoparticle, graphene sheet (guaifenesin)	Chemical crosslinking by epichlorohydrin and ammonium persulfate	- High efficient drug loading and controlled release by pH, external magnetic field, and polymer (starch and alginic acid) contents - Excellent in vivo wound healing potential	[29]
Methacrylate-*g*-high amylose starch (70% of amylose), hydroxyethyl methacrylate-*g*-high amylose starch (theophylline, procaine hydrochloride, bovine serum albumin)	Physical blending	- Controlled release of chemical drugs and proteins by pH and enzyme (α-amylase in pancreatin) - Siutable for hydrophilic matrices for conlon-targeted drug delivery	[30]
Corn starch, di-(1-hydroxyethylene)diselenide (rhodamine B as a model drug)	Chemical crosslinking by potassium persulphate	- Controlled drug release responds to redox agents (dithithreitol, hydrogen peroxide) and enzymes (α-amylase) via cleavage of the diselenide group and starch backbone, respectively	[31]
Carboxymethyl starch (CMS), dextran sulfate (mTHPP)	Physical mixing	- Achieve complex coacervation for the incorporation - Controlled release of an anti-angiogenesis hexapeptide	[32]
Starch, sulfuric acid, gum arabic, K_2_S_2_O_8_	Acid hydrolysis and Physical mixing	- Controlled drug release system - Effectively inhibited cancer cell growth via in vitro anticancer activity	[33]
Iodine, starch, alginate	Ionic cross-linking	- Possesses good photothermal conversion capability - Low cytotoxicity and in vivo toxicity - Powerful tumor suppressing effect	[34]
Starch, poly(*N*-isopropylacrylamide) (Iodine)	Chemical crosslinking (graft copolymerization)	- Simple preparation and good biosafety - Possesses photothermal and anti-infection effect	[35]
Dialdehyde starch, chitosan (betamethasone)	Physical mixing	- Optimized degradation with a fast in vitro and ex vivo release rate - Suited for local administration of betamethasone	[36]
Starch, gelatin	Chemical crosslinking via Schiff base reaction	- Facilitated tissue reconstruction in an in vivo rabbit model - Enhanced smoother skin and no visible scarring	[38]
Corn starch, polyvinyl alcohol, glutaraldehyde	Physical mixing	- increased mechanical strength with addition of polyvinyl alcohol - Reducing the chance of toxicity	[39]
Starch, ammonium propanesulfonate, acrylic acid, PEG, 3-mercaptopropionic acid	Chemical crosslinking via “copper- and light- free” Michael-type “thiol-ene” addition	- A549 cells encapsulated in the hydrogel with high viability - Proliferated and extended after 2 days	[40]
Sulfobetaine derived starch, 3-dimethyl (chloropropyl) ammonium propanesulfonate, methacrylate	Chemical crosslinking via “thiol-ene” Michael addition	- Promoted cell proliferation of encapsulated brown adipose derived stem cells	[41]
Caboxylic starch, dopamine, horseradish peroxidase	Chemical crosslinking (enzymatic crosslinking reaction)	- Faster gelation process and higher hydrogel favorable tissue adhesion property - Possess better hemostatic ability in vitro and in vivo	[42]
Starch, acrylic acid, 2-hydroxy ethyl methacrylate (5-fluorouracil)	Chemical crosslinking via free radical polymerization	- Drug release rate was faster at pH 7.4 that that at pH 1.2 - Porous structure for a useful local delivery system	[56]
Corn starch, ammonium persulfate, pyridine, acryloyl chloride	Chemical crosslinking via radical crosslinking reaction and gas-blowing foaming process	- Improved cell viability, proliferation, and attachment with the incorporation of silk fibroin nanofibers	[57]
Potato starch, silk fibroin, glutaraldehyde	Physical mixing, particulate leaching, freeze drying	- Higher cell viability of osteoblast like cells than pure starch	[58]

## Data Availability

Not applicable.
